# Disease severity as the dominant clinic-available marker of myasthenic crisis occurrence: a single-centre retrospective study

**DOI:** 10.1007/s10072-026-09448-9

**Published:** 2026-10-02

**Authors:** Robert Mîndreanu, Irina Matei, Teodora Stan, Simona Clichici, Dafin Mureșanu, Răzvan Cherecheș, Hanna Dragoș, Alina Blesneag, Delia Stanca, Adina Stan

**Affiliations:** 1 Department of Physiology, Iuliu Hațieganu University of Medicine and Pharmacy, Cluj-Napoca, 400132 Romania; 2 Faculty of Medicine, Iuliu Hațieganu University of Medicine and Pharmacy, Cluj-Napoca, 400132 Romania; 3 Department of Neuroscience, Faculty of Medicine, Iuliu Hațieganu University of Medicine and Pharmacy, Cluj- Napoca, 400132 Romania; 4 HIVE Health Innovation Centre, Babeș-Bolyai University, Cluj-Napoca, 400132 Romania

**Keywords:** Myasthenia gravis, MGFA classification, Mechanical ventilation, Respiratory failure, Outpatient follow-up, Clinical correlates

## Abstract

**Introduction:**

Myasthenic crisis is the most feared acute complication of myasthenia gravis (MG), yet simple clinic-based approaches to risk stratification remain limited. We examined which routinely recorded clinical features were associated with crisis occurrence across the observed disease course and whether a parsimonious model could provide retrospective group-level stratification.

**Methods:**

This single-centre retrospective study included 86 adults with MG. Myasthenic crisis was defined as respiratory failure requiring mechanical ventilation. Disease severity was graded using the Myasthenia Gravis Foundation of America (MGFA) classification and grouped as mild, moderate or severe; MGFA class V was excluded to avoid circularity. Independent correlates were assessed by logistic regression and internally validated by cross-validation. The model was intended for retrospective group-level stratification, not prospective prediction of an individual future crisis.

**Results:**

Thirty-four patients (40%) had experienced a myasthenic crisis. Crisis frequency increased across severity groups: 11%, 26% and 73% in mild, moderate and severe disease, respectively (*p* < 0.001). Each severity grade increased the odds of crisis approximately seven-fold (OR 6.7, 95% CI 2.8–16.5), whereas more frequent outpatient visits were associated with lower odds (OR 0.73). The two-item model showed good discrimination (AUC 0.84; cross-validated AUC 0.79). Calibration-in-the-large was close to ideal, although the cross-validated calibration slope of 0.73 suggested moderate overfitting.

**Conclusions:**

MGFA severity was the dominant clinic-available marker of crisis occurrence. Outpatient-visit frequency contributed less stable information and should be considered exploratory. These findings support retrospective group-level stratification but do not establish prospective prediction or a protective effect of follow-up.

**Supplementary Information:**

The online version contains supplementary material available at https://doi.org/10.1007/s10072-026-09448-9.

## Introduction

Myasthenia gravis (MG) is an autoimmune disorder of the neuromuscular junction characterised by fluctuating, fatigable weakness of skeletal muscle [1]. Its prevalence has risen over recent decades, in part reflecting an ageing population and improved recognition, so that most neurologists now follow a substantial number of affected patients [2, 3]. The most feared acute complication is myasthenic crisis — respiratory failure requiring mechanical ventilation — which affects roughly 15–20% of patients, usually within the first years of disease, and carries considerable morbidity, intensive-care use and cost [4].

Recognised markers of higher crisis risk include a previous crisis, bulbar or oropharyngeal weakness, muscle-specific kinase (MuSK) antibodies, thymoma and intercurrent infection [5, 6]. However, these data derive from cohorts that differ in size and setting and are not easily condensed into a bedside judgement. It remains unclear how well routine clinical findings obtained during a clinically stable assessment capture the broader disease phenotype associated with myasthenic crisis.

We therefore examined, in a consecutive single-centre MG cohort, which routinely available clinical features were associated with myasthenic crisis occurrence across the observed disease course and whether a parsimonious combination of variables could provide clinically interpretable, retrospective risk stratification.

Data on myasthenia gravis from Eastern Europe remain limited, and Romanian cohorts have to date addressed comorbidities [7], HLA associations [8] and treatment experience [9] rather than predictors of myasthenic crisis. To our knowledge, the present series is the first from Romania to examine clinical predictors of myasthenic crisis specifically.

## Methods

### Study design and patients

This was a retrospective, single-centre study conducted at a tertiary neurology department in Romania. Consecutive adults with a neurologist-confirmed diagnosis of MG identified between 1 January 2023 and 1 July 2025 were included (*N* = 89). Diagnosis was supported by compatible clinical findings and available serological and/or neurophysiological evidence. Three patients whose index assessment occurred in MGFA class V were excluded because class V requires intubation, creating direct overlap with the respiratory-support outcome and potential definitional circularity. The final analytic cohort comprised 86 patients.

### Outcome and key definitions

The primary outcome was the documented occurrence of at least one myasthenic crisis during the available disease course, defined as an episode of respiratory failure requiring mechanical ventilation. Disease severity was graded using the MGFA clinical classification [10] and, to keep the analysis clinically interpretable, collapsed a priori into three grades: *mild* (classes I–IIA), *moderate* (IIB–IIIA) and *severe* (IIIB–IVA–IVB). For each patient, the index assessment was defined as the first clinically stable inpatient or outpatient neurological assessment during the study period at which MGFA class could be determined. As the temporal relationship between the stable-state assessment and crisis occurrence varied across patients, the analysis was designed to characterise a crisis-associated clinical risk phenotype and to provide retrospective group-level stratification, rather than to estimate an individual patient’s prospective risk of a future crisis.

The number of scheduled outpatient neurology visits during the observation window was recorded as a simple proxy for engagement with specialist follow-up. Antibody status, thymic pathology, thymectomy and chronic treatments (acetylcholinesterase inhibitors, corticosteroids, azathioprine and other immunosuppressants) were recorded as documented in the chart.

### Statistical analysis

All statistical analysis was performed in Stata v19.5 BE. The final model was deliberately limited to two predictors and its performance assessed by discrimination (area under the receiver-operating-characteristic curve), leave-one-out cross-validation, and calibration (calibration slope and intercept, Hosmer–Lemeshow and Stukel tests [11]); predicted probabilities were additionally recalibrated using cross-validation. Reporting follows the STROBE statement for observational studies [12] (Online Resource 2); because the analysis also yields a multivariable model, discrimination and calibration are additionally reported in line with TRIPOD as a secondary analysis [13]. The available sample met Riley criteria 1 and 2 (small overfitting and minimal shrinkage: required 47 and 44, respectively), but not criterion 3, which governs precise estimation of average absolute risk and required 368 patients [14]. We therefore present the model as an internally validated tool for retrospective risk discrimination and group-level stratification, rather than as a validated instrument for prospective individual prediction. An exploratory decision-curve analysis was performed to examine estimated net benefit across hypothetical threshold probabilities. Because no prospective intervention rule was defined, this analysis was not interpreted as evidence of established clinical utility. Full technical detail, including internal validation, calibration, recalibration, sensitivity analyses and the exploratory decision-curve analysis [15], is provided in Online Resource 1.

## Results

### Cohort characteristics

The cohort comprised 86 patients (median age 60.5 years, IQR 48–72; 44 women, 51%). Disease severity was mild in 18 patients (21%), moderate in 38 (44%) and severe in 30 (35%). Baseline characteristics are shown in Table 1.

**Table 1:** Baseline characteristics of the cohort, overall and by myasthenic crisis occurrence (*N* = 86)

Characteristic	All (*N* = 86)	No crisis (*n* = 52)	Crisis (*n* = 34)	*p*
Demographic and clinical				
Age at data extraction, yr, median (IQR)	60.5 (48–72)	57 (47–69)	69.5 (56–74)	0.051
Female sex, n (%)	44 (51)	24 (46)	20 (59)	0.250
Urban residence, n (%)	62 (72)	38 (73)	24 (71)	0.801
Age at onset, yr, median (IQR) ᵃ	52 (37–66)	46 (37–60)	56 (36–69)	0.157
Disease duration, yr, median (IQR) ᵃ	6 (2–12)	6 (2–12)	5 (2–10)	0.967
Generalised MG, n (%)	77 (90)	43 (83)	34 (100)	0.010
Coexisting autoimmune disease, n (%)	22 (26)	13 (25)	9 (26)	1.000
***MGFA severity***,*** n (%)***				
Mild (I–IIA)	18 (21)	16 (31)	2 (6)	
Moderate (IIB–IIIA)	38 (44)	28 (54)	10 (29)	
Severe (IIIB–IVA–IVB)	30 (35)	8 (15)	22 (65)	< 0.001
***Antibody status***,*** n (%) ᵇ***				
AChR-positive	48 (56)	–	–	
MuSK-positive	5 (6)	–	–	
Anti-titin only	1 (1)			
Seronegative	4 (5)	–	–	
Not documented	28 (33)	–	–	
***Thymus***				
No pathological change, n (%)	53 (62)	–	–	
Hyperplasia, n (%)	12 (14)	–	–	
Thymoma, n (%)	16 (19)	9 (17)	7 (21)	0.778
Thymus not assessed	5(6)			
Thymectomy performed, n (%)	21 (24)	15 (29)	6 (18)	
***Chronic treatment***,*** n (%)***				
Acetylcholinesterase inhibitor	82 (95)	50 (96)	32 (94)	0.646
Corticosteroids	35 (41)	19 (37)	16 (47)	0.332
Azathioprine	37 (43)	18 (35)	19 (56)	0.051
***Outpatient follow-up***				
Outpatient neurology visits, median (IQR)	1 (0–2)	1 (1–3)	0.5 (0–1)	0.002

*IQR*, interquartile range; *MGFA*, Myasthenia Gravis Foundation of America; *AChR*, acetylcholine receptor; *MuSK*, muscle-specific kinase. Continuous variables compared with the Mann–Whitney U test; categorical variables with the chi-square or Fisher exact test. ^a^Available in 78 patients. ^b^AChR-positive includes 2 patients with concurrent anti-titin antibodies

### Myasthenic crisis and its relation to severity

Thirty-four patients (40%) had experienced at least one myasthenic crisis. Crisis frequency increased sharply across severity grades — 11% (2/18) in mild, 26% (10/38) in moderate and 73% (22/30) in severe disease (*p* < 0.001). Every crisis occurred in a patient with generalised disease (0 of 9 purely ocular patients).

### Features associated with crisis

**Fig. 1:**
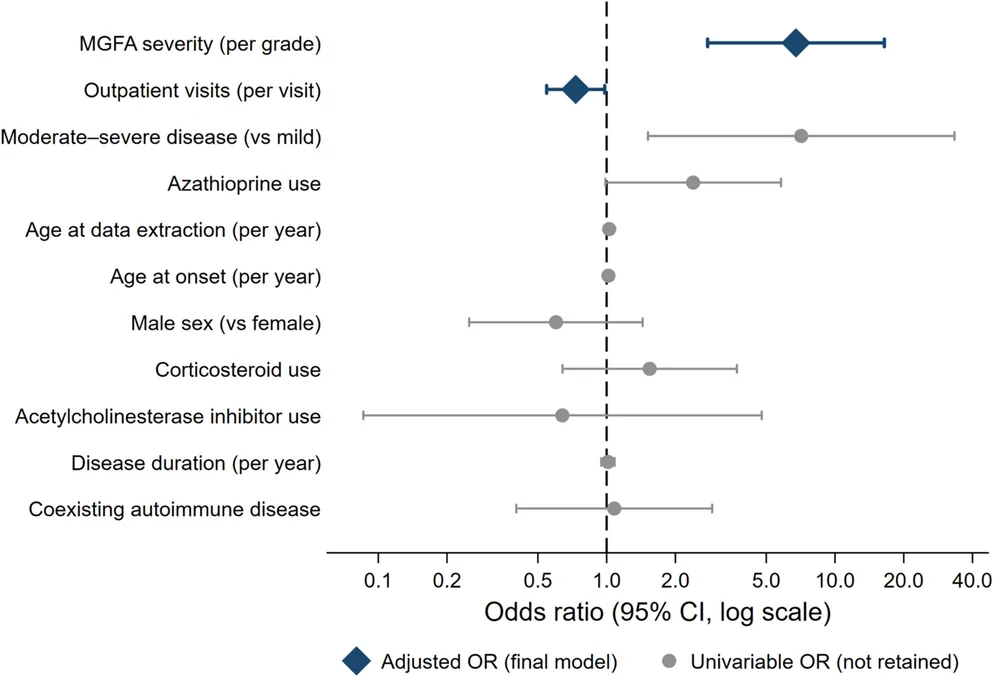
Forest plot of clinical features associated with documented myasthenic crisis occurrence across the observed disease course. Diamonds represent adjusted odds ratios (ORs) with 95% confidence intervals (CIs) for the two variables retained in the final multivariable model; circles represent univariable ORs with 95% CIs for candidate variables not retained in the final model. MGFA severity was modelled ordinally per one-grade increase (mild to moderate or moderate to severe), and outpatient visits per additional scheduled visit during the study period. The vertical dashed line denotes an OR of 1. MGFA, Myasthenia Gravis Foundation of America; OR, odds ratio; CI, confidence interval

On single-variable analysis, only disease severity was clearly associated with crisis (Table 2; Fig. 1); more frequent outpatient visits showed an inverse association that reached significance only after adjustment (Table 3). No other feature — including sex, residence, disease duration, age at onset, thymoma, coexisting autoimmune disease or chronic treatments — was associated with crisis.

**Table 2:** Single-variable association of clinical features with myasthenic crisis (*N* = 86)

Feature	Odds ratio (95% CI)	*p*
MGFA severity (per grade)	5.65 (2.50–12.81)	< 0.001
Moderate–severe disease (vs. mild)	7.11 (1.52–33.34)	0.013
Azathioprine use	2.39 (0.99–5.80)	0.054
Age at data extraction (per year)	1.03 (1.00–1.06)	0.077
Outpatient visits^a^ (per visit)	0.78 (0.58–1.05)	0.097
Age at onset (per year)	1.02 (0.99–1.05)	0.176
Male sex (vs. female)	0.60 (0.25–1.44)	0.252
Corticosteroid use	1.54 (0.64–3.72)	0.333
Acetylcholinesterase inhibitor use	0.64 (0.09–4.77)	0.663
Disease duration (per year)	1.01 (0.95–1.08)	0.695
Coexisting autoimmune disease	1.08 (0.40–2.90)	0.879

^a^Outpatient visit counts were concentrated at low values (median 1, IQR 0–2; Table 1). The Mann–Whitney test in Table 1 compares the distribution of visits between groups, whereas univariable logistic regression estimates a constant change in log-odds per additional visit; the two analyses therefore address different questions. The inverse association strengthened after adjustment for MGFA severity (Table 3). CI, confidence interval; MGFA, Myasthenia Gravis Foundation of America. Each row is a separate univariable logistic regression

**Table 3:** Final model for myasthenic crisis: single-variable versus adjusted estimates

Feature	Univariable OR (95% CI)	Adjusted OR (95% CI)	*p*
MGFA severity (per grade)	5.65 (2.50–12.81)	6.75 (2.77–16.45)	< 0.001
Outpatient visits (per visit)	0.78 (0.58–1.05)	0.73 (0.55–0.98)	0.037

OR, odds ratio; CI, confidence interval. Model discrimination: area under the curve 0.84 (95% CI 0.75–0.93); cross-validated 0.79. Adding the number of outpatient visits to severity improved model fit (Akaike information criterion 95.9 → 92.4)

### Retrospective risk stratification using a two-item clinical model

In the final model, both MGFA severity and the number of outpatient visits were independently associated with crisis occurrence across the observed disease course. Higher severity markedly increased the odds of crisis, whereas a larger number of recorded outpatient visits showed an inverse association (Online Resource 1, Table S1). The model showed good discrimination, with an apparent AUC of 0.84 and a leave-one-out cross-validated AUC of 0.79 (Online Resource 1, Fig. S2). Calibration-in-the-large was close to ideal, whereas the cross-validated calibration slope of 0.73 indicated moderate overfitting and some overestimation of risk at the extremes (Online Resource 1, Fig. S1a).

**Fig. 2:**
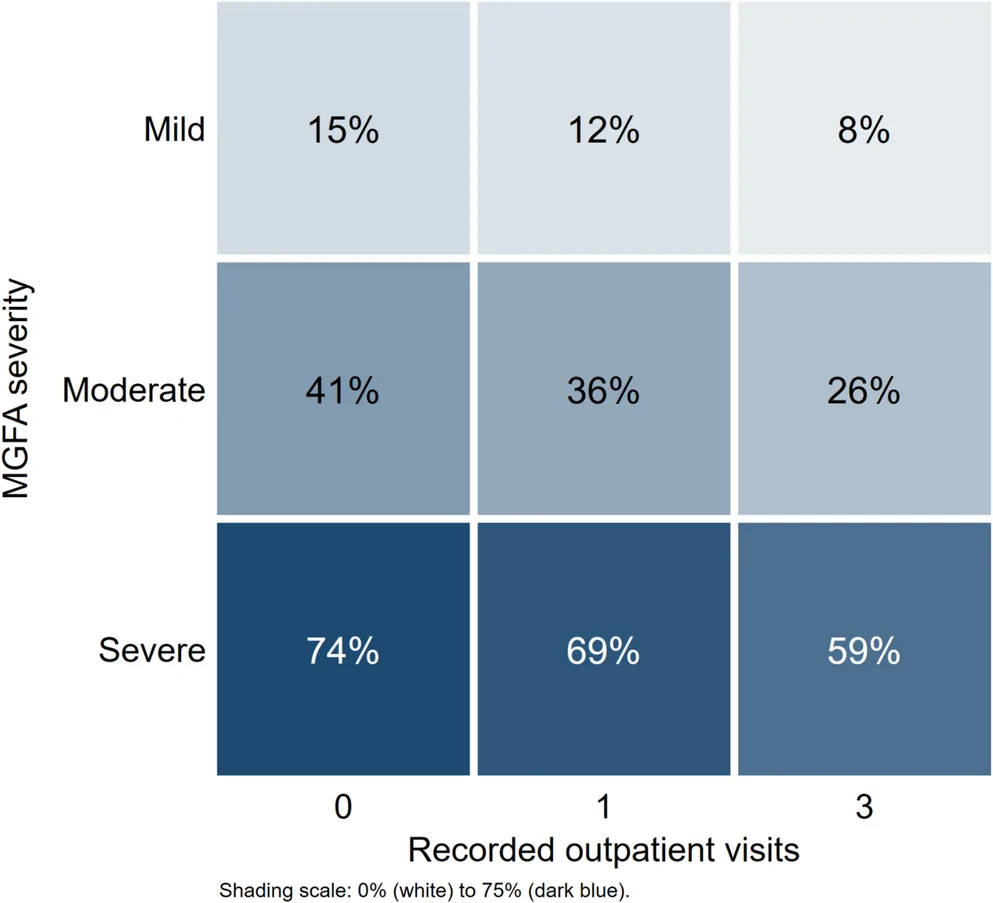
Model-estimated probability of documented myasthenic crisis occurrence across the observed disease course, by MGFA severity and outpatient-visit frequency (0, 1 or 3 recorded visits; columns are not equally spaced). Each cell is the model-estimated probability of crisis for a patient in that severity–follow-up stratum (cross-validation-recalibrated). Values are percentages, not patient counts; cells are independent estimates and are not additive — they do not sum to the cohort size. Apparent (non-recalibrated) probabilities were broadly similar (mild 9/7/4%, moderate 40/33/21%, severe 82/77/64% for 0/1/3 visits). These are retrospective, within-cohort group estimates and should not be interpreted as prospective probabilities of a future crisis

In the cross-validation–recalibrated within-cohort estimates shown in Fig. 2, the model-estimated probability of documented crisis occurrence ranged from approximately 8% among patients with mild disease and three recorded outpatient visits to 74% among patients with severe disease and no recorded outpatient visits. Across sensitivity analyses (Online Resource 1, Table S1), disease severity remained the dominant and stable predictor; the outpatient-visit association was less stable, attenuating to the null among patients with at least one admission (OR 0.94) and varying in magnitude across subgroups.

## Discussion

In this consecutive tertiary-centre MG cohort, stable-state MGFA severity mapped onto a steep gradient of myasthenic crisis occurrence across the observed disease course. The odds increased approximately seven-fold with each severity grade, and almost three-quarters of patients in the severe category had experienced a crisis. The two-item model provided good internally validated discrimination between lower- and higher-risk clinical strata. These findings support MGFA severity as a pragmatic marker of a crisis-prone disease phenotype, while not establishing prospective prediction of a future event. This is consistent with the view that crisis is largely a manifestation of more severe disease [5, 16].

Previously reported predictors — disease severity, MuSK antibodies, thymoma, bulbar weakness, prior crisis and infection — derive from cohorts that differ in size, setting and data source, including multicentre hospital-based, single-centre and claims-based studies [5, 6, 17]. Our clinic-based finding that overall severity dominates is compatible with these data and has the advantage of using a grade that neurologists already assign at every visit. We did not find thymoma associated with crisis, most likely because of the small number of affected patients.

A notable observation was that every documented crisis had occurred in a patient with generalised disease, with none among the nine patients with purely ocular involvement. This is consistent with the nature of myasthenic crisis, which is defined by respiratory and bulbar muscle failure and therefore does not arise in disease confined to the extraocular muscles. Clinically, it reinforces that generalisation is effectively a prerequisite for crisis: patients with purely ocular disease have a very low likelihood of crisis, whereas the transition to generalised MG marks entry into the at-risk group that warrants closer attention.

The inverse association with outpatient follow-up warrants caution. It can be read in two ways. It may be genuinely protective: regular specialist review allows earlier detection of deterioration and timely escalation of immunotherapy [16, 18, 19]. Equally, it may reflect reverse causation and ascertainment — patients who deteriorate early or abruptly present acutely and are admitted rather than attending scheduled clinics [20], and a lifetime history of crisis was compared with visits counted over a defined window. We therefore present this finding as hypothesis-generating rather than as evidence that additional appointments prevent crises. Its practical value is to flag patients who are disengaged from care as a group that deserves closer attention.

Greater disease severity has already been associated with myasthenic crisis: severity at diagnosis was an independent predictor of crisis or disease exacerbation in a multicentre cohort of 815 patients [5], and crisis risk increased across MGFA classes at diagnosis in a single-centre cohort of 1150 patients analysed with time-to-event methods [21]. The contribution of the present study therefore lies not in the association itself, but in three complementary elements: data from an under-represented Eastern European population, representing, to our knowledge, the first Romanian series to examine clinical predictors of myasthenic crisis; severity graded at a clinically stable assessment rather than at diagnosis, although crisis may have preceded this assessment; and a two-item stratification model with internal validation, in which outpatient-visit frequency remained exploratory. A simple clinic-based assessment captured most of the observed separation between lower- and higher-risk strata, without requiring specialised biomarkers or additional instruments. The model should be viewed as a parsimonious risk-stratification framework and a basis for prospective validation, rather than as a ready-to-use individual prediction tool. The exploratory decision-curve analysis is hypothesis-generating only and does not establish prospective clinical benefit or justify model-guided treatment decisions.

Several limitations should be acknowledged. The study is retrospective and single-centre. Both crisis and severity were ascertained from the lifetime clinical record, so temporal precedence cannot be guaranteed and some residual circularity is possible despite the exclusion of MGFA class V. With 34 events, confidence intervals are wide and subgroups such as thymoma and MuSK-positive patients are underpowered; antibody status was undocumented in about a third of patients. The window over which outpatient visits were counted may not coincide with the timing of crisis. Disease severity was captured only by the MGFA classification; validated MG-specific instruments such as the MG-ADL, QMG and MGC scores were not consistently available in this retrospective cohort, and their incorporation could refine risk discrimination in future prospective studies. Accordingly, the model provides retrospective risk discrimination and identifies a crisis-associated clinical phenotype, but it does not establish temporal causality or validated prospective prediction. External prospective evaluation is required before individual future-risk estimates or model-guided clinical decisions can be considered.

## Conclusion

In this tertiary-centre MG cohort, stable-state MGFA severity was the dominant clinic-available marker of a crisis-prone disease phenotype and supported clear retrospective, group-level risk stratification. The addition of outpatient-visit frequency modestly improved model fit, although this association was less stable and should be interpreted as exploratory. The model is not a validated predictor of an individual future crisis, but provides a parsimonious framework for prospective evaluation in larger multicentre cohorts.

## Supplementary Information

Below is the link to the electronic supplementary material.

Online Resource 1

Online Resource 2

## Data Availability

The analytic dataset and full statistical code are available upon reasonable request.
