# Supplementary material for: Disease severity as the dominant clinic-available marker of myasthenic crisis occurrence: a single-centre retrospective study
Source: Neurol Sci. 2026 Oct 2;47(10):830. doi: 10.1007/s10072-026-09448-9 (PMC13633172; doi:10.1007/s10072-026-09448-9)
Supplement: Supplementary file 1 [file 10072_2026_9448_MOESM1_ESM.docx]

Online Resource 1 — Supplementary figures and tables

Manuscript: “Disease severity as the dominant clinic-available marker of myasthenic crisis occurrence: a single-centre retrospective study”

Journal: Neurological Sciences

Supplementary Figure S1. Model calibration and exploratory decision-curve analysis

**a**

**b**

**c**

**Fig. S1** Model calibration and exploratory decision-curve analysis. (a) Calibration of the two-item model under leave-one-out cross-validation: grouped observed versus expected crisis frequency (95% CI) with a locally weighted smoother; O:E 1.003, calibration-in-the-large 0.007, calibration slope 0.732, cross-validated AUC 0.791; Hosmer–Lemeshow test (eight requested groups, six distinct because of tied predicted probabilities) χ²(4) = 5.61, p = 0.231; Stukel test χ²(2) = 0.31, p = 0.855. (b) Cross-validation recalibration: apparent versus LOOCV-recalibrated predicted probabilities (slope 0.73). (c) Exploratory decision-curve analysis with 95% bootstrap confidence intervals, showing estimated net benefit under hypothetical threshold probabilities of 0.1–0.6. No specific prospective intervention rule was defined, and the analysis should not be interpreted as establishing clinical utility.

Supplementary Figure S2. Discrimination (ROC)

**Fig. S2** Receiver-operating-characteristic curves for the two-item model, showing apparent (AUC 0.84) and leave-one-out cross-validated (AUC 0.79) discrimination

Supplementary Table S1. Sensitivity analyses — stability of the two-item model

| **Analysis** | **n** | **MGFA severity OR (95% CI)** | **Visits OR (95% CI)** | **AUC** |
| --- | --- | --- | --- | --- |
| Main model | 86 | 6.75 (2.77–16.45) | 0.73 (0.55–0.98) | 0.84 |
| Excluding in-hospital death | 85 | 6.51 (2.67–15.86) | 0.74 (0.55–0.99) | 0.83 |
| Adjusted for age at onset | 78 | 6.97 (2.73–17.78) | 0.79 (0.59–1.06) | 0.83 |
| Patients with ≥1 admission | 53 | 5.47 (2.01–14.90) | 0.94 (0.66–1.32) | 0.79 |
| Excluding severe disease | 56 | 3.36 (0.53–21.05) | 0.20 (0.06–0.67) | 0.82 |
| Severity only (single predictor) | 86 | 5.65 (2.50–12.81) | – | 0.78 |

OR, odds ratio; CI, confidence interval; AUC, area under the receiver-operating-characteristic curve. “Excluding in-hospital death” omits the one patient who died during the last hospital admission. Disease severity remained the dominant predictor in all analyses except the exclusion of patients with severe disease (n = 56), where its estimate retained the same direction but was imprecise (95% CI 0.53–21.05); the outpatient-visit association was less stable, attenuating to the null among patients with at least one admission.
