# Supplementary material for: Disease severity as the dominant clinic-available marker of myasthenic crisis occurrence: a single-centre retrospective study
Source: Neurol Sci. 2026 Oct 2;47(10):830. doi: 10.1007/s10072-026-09448-9 (PMC13633172; doi:10.1007/s10072-026-09448-9)
Supplement: Supplementary file 2 [file 10072_2026_9448_MOESM2_ESM.docx]

Online Resource 2 — STROBE

Manuscript: “Disease severity as the dominant clinic-available marker of myasthenic crisis occurrence: a single-centre retrospective study”

Journal: Neurological Sciences

Supplementary Figure S3. Study flow diagram (STROBE)

**Fig. S3** Flow of participants. Of 89 consecutive adults with confirmed myasthenia gravis, three patients whose index assessment occurred in MGFA class V were excluded because class V requires intubation, creating direct overlap with the respiratory-support outcome and potential definitional circularity

STROBE Statement — checklist of items for cross-sectional / cohort studies

| **#** | **STROBE item** | **Location in manuscript** |
| --- | --- | --- |
| 1 | Title & abstract | Title; Abstract |
| 2 | Background / rationale | Introduction, paras 1–2 |
| 3 | Objectives | Introduction, para 3; Abstract |
| 4 | Study design | Methods 2.1; Abstract |
| 5 | Setting, locations, dates | Methods 2.1 |
| 6 | Participants — eligibility, selection | Methods 2.1; Fig. S3 |
| 7 | Variables | Methods 2.2 |
| 8 | Data sources / measurement | Methods 2.1–2.2 |
| 9 | Bias | Discussion (limitations); Methods 2.2 |
| 10 | Study size | Methods 2.1; 2.3 (Riley) |
| 11 | Quantitative variables | Methods 2.2 |
| 12 | Statistical methods | Methods 2.3 |
| 13 | Participants — numbers per stage | Results 3.1; Fig. S3 |
| 14 | Descriptive data | Results 3.1; Table 1 |
| 15 | Outcome data | Results 3.2; Table 1 |
| 16 | Main results | Results 3.3–3.4; Tables 2–3 |
| 17 | Other analyses | Results 3.4; Fig. 2; Table S1 |
| 18 | Key results | Discussion; Conclusion |
| 19 | Limitations | Discussion (limitations) |
| 20 | Interpretation | Discussion |
| 21 | Generalisability | Discussion |
| 22 | Funding | Declarations |

Adapted from von Elm E, Altman DG, Egger M, Pocock SJ, Gøtzsche PC, Vandenbroucke JP. The STROBE statement. Lancet 2007;370:1453–1457.
